# Therapeutic potential of anticoagulant therapy in association with cytokine storm inhibition in severe cases of COVID-19: A case report

**DOI:** 10.1515/biol-2021-0088

**Published:** 2021-08-23

**Authors:** Qiancheng Xu, Tao Wang, Weihua Lu

**Affiliations:** Department of Critical Care Medicine, The First Affiliated Hospital of Wannan Medical College (Yijishan Hospital of Wannan Medical College), No. 2, West Road of Zheshan, Jinghu District, Wuhu, Anhui, 241000, China

**Keywords:** COVID-19, SARS-CoV-2, tocilizumab, cytokine storm, inflammation, coagulation

## Abstract

Inflammation and coagulation are considered to the development of Coronavirus disease 2019 (COVID-19)-related hypoxemia. However, this is still controversial, which brings challenges to clinical treatment. Here, we reviewed the levels of interleukin-6 (IL-6), coagulation indexes, and clinical manifestations of a patient with severe COVID-19 after Tocilizumab administration. In this case, the patient’s body temperature quickly dropped to normal after using Tocilizumab, while C reactive protein progressively decreased and stabilized at a lower level. However, IL-6 and D-dimers increased and were accompanied by a continuous decrease of the oxygenation index. After anticoagulant therapy with heparin, D-dimer decreased slowly, gradually improving the oxygenation index and disease remission. This case suggests that the formation of microthrombus might be the main reason for COVID-19-derived hypoxemia. However, the mechanism of hypoxemia and the role of Tocilizumab in COVID-19 need further research. Nevertheless, these findings might still assist medical workers in formulating timely treatment strategies for similar severe patients.

## 1 Background

Coronavirus disease 2019 (COVID-19) has been a global epidemic for nearly 2 years since its emergence in China in late 2019 [1]. However, it is still unclear if inflammation and coagulation are relevant to COVID-19-related hypoxemia [2,3], which brings challenges to clinical treatment. Tocilizumab, an IL-6 receptor blocker, is considered to be an effective treatment for COVID-19, but after recent randomized controlled trials, no definitive conclusions have been drawn [4,5]. However, IL-6 initiates the systemic pro-inflammatory response and inhibits inflammation, thus promoting cell proliferation and tissue repair [6]. Moreover, inappropriate routine use of Tocilizumab to treat COVID-19 should not be encouraged until there is insufficient evidence. Therefore, we measured IL-6, coagulation indexes, and clinical manifestations in a severe COVID-19 patient after the use of Tocilizumab (in accordance with the regulations of the National Health Commission of China) [7]. This study aimed to better understand the pathophysiology of COVID-19-related hypoxemia and improve the clinical strategy for the treatment of this disease.

## 2 Case report

**Figure 1:**
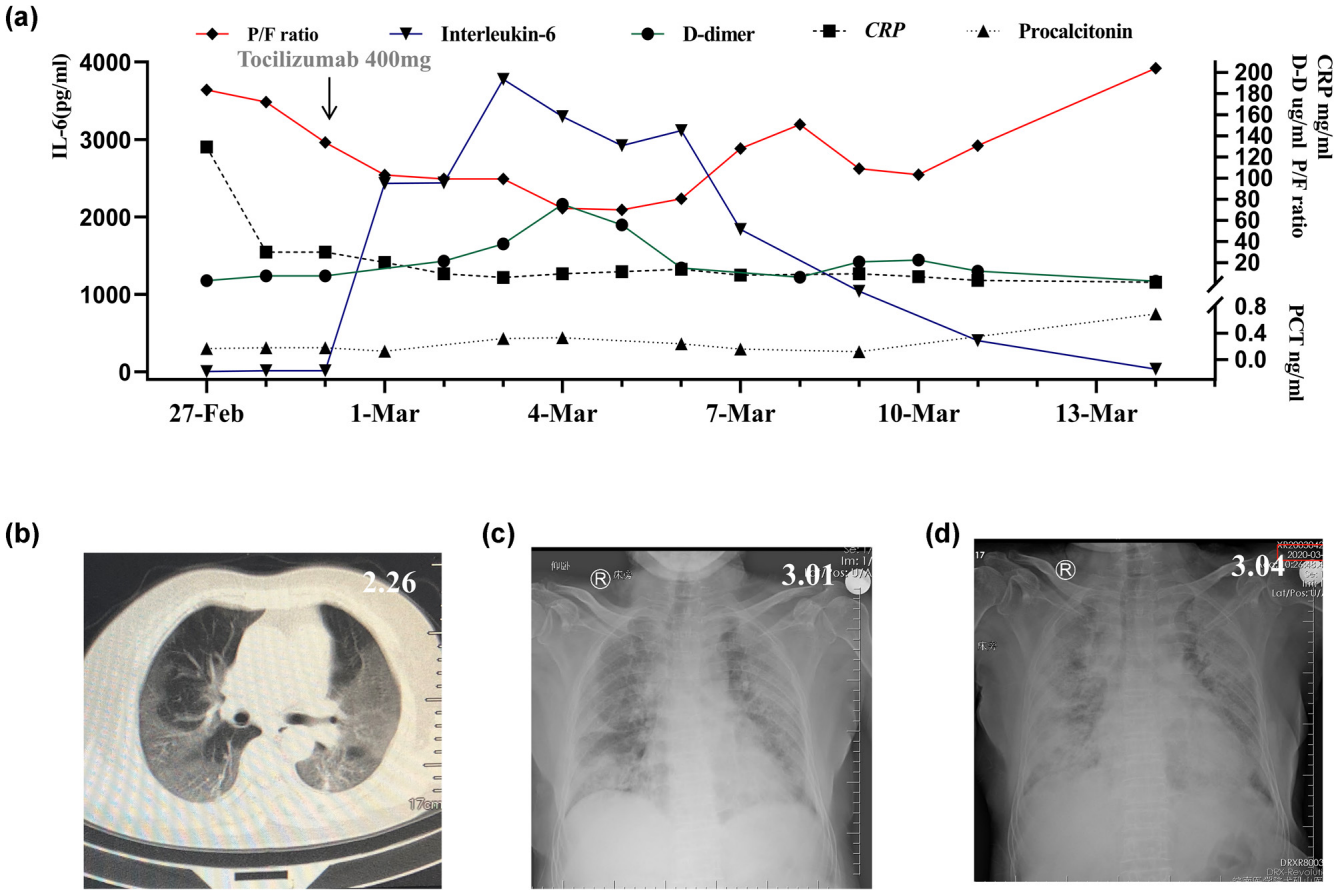
Changes in FiO_2_, P/F ratio (a), IL-6, CRP, D-dimer, PCT in a severe COVID-19 patient (a). Chest CT showed multiple shadows in both lungs on February 26 (b). X-ray showed multiple infiltrations in both lungs on March 01 (c). X-ray showed more severe infiltration 3 days before on March 04 (d). FiO_2_: fraction of inspiration O_2_; P/F ratio: SpO_2_/FiO_2_ ratio; IL-6: interleukin-6; CRP: C reactive protein; PCT: procalcitonin.

On February 27, 2020, a 65-year-old woman was admitted to our fever clinic with symptoms of fever, cough, fatigue, and shortness of breath. She had developed fever 6 days back with a maximum temperature of 37.8°C. She did not present cold and chills before the fever. The cough started 4 days before with a small amount of white phlegm (mucus), no chest tightness, chest pain, or dyspnea. Apparent chest tightness and dyspnea began to appear on February 27, 2020, with SpO_2_ of 80%; Chest CT showed multiple shadows in both lungs (Figure 1b); leukocyte count was 6.58 × 10^9^/L, absolute lymphocyte count was 0.44 × 10^9^/L and C reactive protein (CRP) was 97.3 mg/L. The patient denied that she had been to Wuhan but had a history of contact with asymptomatic people who had returned from the COVID-19 epidemic area in Wuhan.

The patient was diagnosed with suspected COVID-19 virus infection and was immediately admitted to the isolation ward and kept at the prone position combined with high-flow oxygen therapy. Interferon α-2b (5 million units twice daily, atomization inhalation) and Arbidol (200 mg twice daily, orally) were prescribed as antiviral therapy, and moxifloxacin (0.4 g once daily, intravenously) was used to prevent secondary bacterial infections. Methylprednisolone (40 mg twice) was administered to attenuate the lung inflammation and a throat swab sample was taken (Table 1). Laboratory test results are listed in Table 2. On February 28, 2020 (Day 8 after the beginning of symptoms and second day of hospitalization), COVID-19 was confirmed by reverse transcription PCR performed by the Anhui Provincial Centers for Disease Control (CDC).

**Table 1:** Timeline of disease course according to days from initial presentation of illness and days from hospital admission

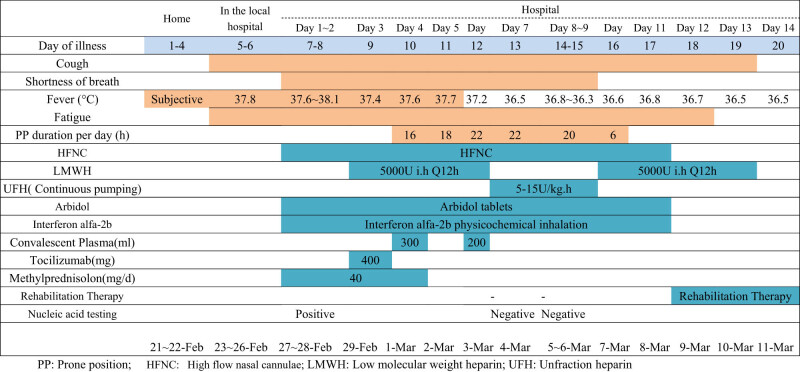

**Table 2:** Clinical laboratory tests

	Hospital Day	Day 1	Day 3	Day 4	Day 6	Day 8	Day 10	Day 11	Fever clinic
	Illness Day	7	9	10	12	14	16	17	36
Measure	Reference range	Date							
		27-Feb	29-Feb	1-Mar	3-Mar	5-Mar	7-Mar	8-Mar	29-Mar
Complete blood count									
White-cell count (×10^9^/L)	4–10	2.9	7	5.4	5.3	9.3	10.3	11	8.7
Absolute neutrophil count (×10^9^/L)	2.0–7.5	2.4	6.4	4.2	4.6	8.2	8.4	8.5	6.3
Absolute Lymphocyte count (×10^9^/L)	2.0–7.5	0.4	0.4	1.1	0.6	0.7	0.8	0.9	2
Absolute monocyte count (×10^9^/L)	0.12–0.8	0.1	0.1	0.1	0.6	0.3	0.3	0.3	0.4
Red-cell count (×10^12^/L)	3.5–5.0	3.76	3.73	3.58	3.75	3.57	3.76	3.99	3.62
Hemoglobin (g/L)	110–150	118	116	110	116	113	121	123	119
Platelet count (×10^9^/L)	100–300	168	207	215	217	212	218	221	280
Biochemical test									
Total protein (g/L)	65.0–85.0	56.8	73.4	69.9	73.5	75.6	74.3	73.9	64.1
Albumin (g/L)	40.0–55.0	29.8	33.5	32	33.9	41.1	40.6	39.7	32.1
Prealbumin (mg/L)									
Alanine aminotransferase (ALT) (U/L)	7–40	44	78	55	43	31	36	32	19
Aspartate aminotransferase (AST) (U/L)	13–35	76	50	35	30	39	41	37	16
Lactate dehydrogenase (LDH) (U/L)	135–225	438	343	328	388	559	561	511	213
Urea (mmol/L)	2.3–7.1	3.5	6.8	6.4	5.9	4.6	4.9	4.8	2.28
Creatinine (µmol/L)	40–130	43.4	49.8	52.3	39	43.9	44.6	44.3	41.8
Sodium (mmol/L)	135–149	139.2	143.7	142.8	140.2	140	141	140.7	139.4
Potassium (mmol/L)	3.5–5.3	3.87	3.53	3.22	3.36	3.84	3.76	4.02	4.64
Chloride (mmol/L)	99–110	101.9	101.8	102.9	102	97.8	102.1	101.5	102
Arterial blood gas (ABG) analysis									
pH	7.350–7.450	7.443	7.452	7.5	7.477	7.499	7.456	7.433	
Pressure of oxygen in arterial blood (mm Hg)	83–108	76.4	80.3	61.8	79.4	63.1	89.7	98	
Pressure of carbon dioxide in arterial blood (mm Hg)	35–45	29.4	33.6	33.2	29.6	34.7	38.9	44.7	
Base excess (mmol/L)	−3 to 3	−3.6	−0.4	2.6	−1.5	3.6	3.3	5.2	
Coagulation profile									
Prothrombin time (s)	11–14.5	12.8	12.5	12.3	11.2	13.1		12.1	12.6
International normalized ratio	0.8–1.25	1.11	1.08	1.06	0.96	1.13		1.04	1.09
Fibrinogen (g/L)	1.8–4.0	6.04	4	3.24	1.99	2.43		4.57	4.33

The patient’s oxygenation index decreased gradually between the 1st and 3rd days of hospitalization. On February 29, 2020, the patient presented with a high-flow nasal cannula (HFNC) oxygen therapy (60% concentration, flow rate: 40 L/min) and partial pressure of oxygen of 80.3 mm Hg. A chest X-ray showed progressive infiltrate and diffuse gridding shadows in both lungs (Figure 1c and d). Tocilizumab 400 mg was administered intravenously. The oxygenation index between the 3rd and 8th days of hospitalization progressively decreased, while IL-6 rapidly increased to 3,775 pg/mL, and then started to slowly decrease. CRP and procalcitonin (PCT) remained at a relatively low level, but the D-dimer gradually increased, showing the same trend as the decrease of the oxygenation index. Heparin anticoagulation therapy was given on the 3rd day of hospitalization (Table 1), D-dimer gradually decreased on the 7th day of hospitalization, and the oxygenation index gradually improved on the 8th day of hospitalization. A throat swab nucleic acid detection was negative for COVID-19 on the 9th day of hospitalization (Figure 1a). On March 9, the patient was transferred to a local hospital.

**Informed consent:** Informed consent has been obtained from all individuals included in this study.**Ethical approval:** The research related to human use has been complied with all the relevant national regulations, institutional policies and in accordance with the tenets of the Helsinki Declaration, and has been approved by the authors’ institutional review board or equivalent committee.

## 3 Discussion

The dysregulation of host immune response characterized by a cytokine storm and lymphocyte depletion is a prominent manifestation of SARS-CoV-2 infection. Critically ill patients with COVID-19 are often accompanied by a significant increase in IL-6 and inflammatory cytokine storm, which are considered key factors that lead to a rapid progression of the disease and culminating in death [8]. China’s “Diagnosis and Treatment Protocol for Novel Coronavirus Pneumonia (Trial Version 7)” suggests that tocilizumab can be used for severe patients with increased IL-6 [7]. Tocilizumab is a monoclonal antibody against the IL-6 receptor (IL-6R). At present, this drug has been successfully used to treat a variety of chronic inflammatory diseases, such as rheumatoid arthritis, systemic and polyarticular juvenile idiopathic arthritis, and Castleman’s disease [9]. However, so far, there is no definitive evidence that Tocilizumab is effective against severe cases of COVID-19, while the relevance of the inflammatory cytokine storm in COVID-19 is still controversial [2,3,10].

In the case herein presented, the patient’s body temperature quickly dropped to normal after the use of tocilizumab; however, the respiratory function did not improve. The oxygenation index continued to decrease until March 5 before slowly increasing. Chest CT showed that bilateral lung exudation was still progressing gradually. Levels of IL-6 (pg/mL) and D-dimer (μg/mL) were increased later (from baseline of 3.1 and 3.0 to 3,114.0 and 73.70, respectively). This is similar to two cases previously reported in Chest. After tocilizumab use, IL-6 (pg/mL) and D-dimer (ng/mL) increased from 74.3 and 982 to 345 and 30,233, respectively [11].

Qin et al. [12] reported a COVID-19 patient with an IL-6 of 25 pg/mL and an acute respiratory distress syndrome(ARDS) of up to 1,618 pg/mL, which is ten times higher than that of other reported cases of COVID-19 [13]. Another study published in JAMA came to the same conclusion [14]. Besides, there are obvious differences with traditional ARDS in lung compliance and ventilation blood flow [10]. Therefore, it is suggested that the formation of microthrombus was the main reason for the decrease of oxygenation index in our reported case.

A growing number of studies have shown that the main cause of hypoxemia in COVID-19 patients is due to vasodilation that occurs upon the activation of vascular endothelial cells. This may result in pulmonary microvascular embolism; however, the effect of inflammatory factors is not obvious [7,11]. An autopsy result confirmed that there was no significant inflammatory cell infiltration in the renal interstitium despite the activation of endothelial cells and the detection of virus particles [15]. Zheng et al. [16] suggested that D-dimer is an important predictor of the prognosis of COVID-19 patients. This conclusion has been confirmed in other studies [6]. Therefore, anticoagulant therapy is considered to be an important strategy to improve the prognosis of patients with COVID-19 [2,17,18]. In our reported case, D-dimer continued to increase and was accompanied by a continuous decrease of the oxygenation index after the application of tocilizumab. After anticoagulant therapy with heparin, D-dimer decreased slowly, and the oxygenation index gradually improved. D-dimer had a significant negative correlation with the oxygenation index of the patient, suggesting that the activation of vascular endothelial cells and the formation of microthrombus were the main causes for the decrease of oxygenation index in this patient.

In this case, it was not clear whether there is a causal relationship between the use of tocilizumab and the increase of D-dimer. Previous studies have shown that IL-6 can exert different activities (pro-inflammatory and anti-inflammatory) through classic- and trans-signaling pathways [9,19]. Under steady-state conditions, the IL-6 buffer in the blood can prevent systemic IL-6 activity. Only when the blood level of IL-6 is strongly elevated and exceeds that of soluble IL-6 receptor (sIL-6R) can the trans-signaling pathway of IL-6 and systemic inflammation be activated [20,21]. Previous studies have shown that genetic deletion of the IL-6 receptor does not improve the prognosis of septic mice, while the application of soluble gp130-Fc can significantly reduce the survival rate of CLP mice, decreasing apoptosis of endothelial cells [22]. In addition, the cytokine storm contains a variety of other inflammatory factors (IL-8, IL-10, IFN-γ and MCP-1, TNF-α, IL-1, and IL-3) [23]. Blocking IL-6R with tocilizumab alone may be insufficient to improve the prognosis of COVID-19 patients.

The reported case suggests that the formation of microthrombus might be the main reason for hypoxemia; however, the mechanism of hypoxemia and the role of tocilizumab in COVID-19 need further research. Nevertheless, these findings may still assist medical workers to formulate timely treatment strategies for similarly severe patients.
